# The dietary proportion of essential amino acids and Sir2 influence lifespan in the honeybee

**DOI:** 10.1007/s11357-014-9649-9

**Published:** 2014-04-10

**Authors:** Pier P. Paoli, Luisa A. Wakeling, Geraldine A. Wright, Dianne Ford

**Affiliations:** 1 Institute of Neuroscience, Newcastle University, Medical School, Newcastle upon Tyne, NE2 4HH UK; 2 Institute for Cell and Molecular Biosciences and Institute for Ageing and Health, Newcastle University, Medical School, Newcastle upon Tyne, NE2 4HH UK

**Keywords:** Honeybees, Sir2, Diet, Protein, Essential amino acids, Lifespan

## Abstract

Dietary essential amino acids have an important influence on the lifespan and fitness of animals. The expression of the NAD^+^-dependent histone deacetylase, Sir2, can be influenced by diet, but its role in the extension of lifespan has recently been challenged. Here, we used the honeybee to test how the dietary balance of carbohydrates and essential amino acids and/or Sir2 affected lifespan. Using liquid diets varying in their ratio of essential amino acids to carbohydrate (EAA:C), we found that adult worker bees fed diets high in essential amino acids (≥1:10) had shorter lifespans than bees fed diets containing low levels of dietary amino acids. Bees fed a 1:500 EAA:C diet lived longer and, in contrast to bees fed any of the other diets, expressed Sir2 at levels tenfold higher or more than bees fed a 1:5 EAA:C diet. When bees were fed the 1:500 diet, small interfering RNA (siRNA)-mediated knock-down of Sir2 expression shortened lifespan but did not reduce survival to the same extent as the 1:5 diet, indicating that Sir2 contributes to mechanisms that determine lifespan in response to differences in macronutrient intake but is not the sole determinant. These data show that the ratio of dietary amino acids to carbohydrate influences Sir2 expression and clearly demonstrate that Sir2 is one of the factors that can determine honeybee lifespan. We propose that effects of dietary amino acids and Sir2 on lifespan may depend on the simultaneous activation of multiple nutrient sensors that respond to relative levels of essential amino acids and carbohydrates.

**Electronic supplementary material:**

The online version of this article (doi:10.1007/s11357-014-9649-9) contains supplementary material, which is available to authorized users.

## Introduction

The quantity and quality of food an animal eats directly affects its lifespan and fitness. An early study in rats that revealed an effect of caloric intake on longevity (McCay et al. 1935) has since sparked investigations on the effect of caloric restriction on lifespan in animals as diverse as yeast and humans (Guarente 2005; Meydani et al. 2011). However, dietary restriction in the form of a reduced intake of total dietary macronutrients does not always produce a longer-lived phenotype, as shown, for example, by recent discordant observations in two studies in Rhesus monkeys (Colman et al. 2009; Mattison et al. 2012). Elevated levels of certain amino acids (Grandison et al. 2009) and protein (Altaye et al. 2010; Lagiou et al. 2007) can reduce survival. It is thus unclear whether the effect of diet on lifespan is a function of energy intake, dietary amino acids and proteins, or both. Indeed, the dietary balance of specific nutrients rather than total intake may be the factor that influences lifespan (Levine et al. 2014; Solon-Biet et al. 2014; reviewed in Simpson and Raubenheimer 2012; Tatar 2011; Piper et al. 2011).

The expression of sirtuins—notably SIRT1 in mammals and its homologue in other species (Sir2 in yeast and *Drosophila*, sir-2.1 in *Caenorhabditis elegans*; for simplicity referred to hereafter using the generic term Sir2)—has been linked to extended lifespan and is likely to play a key role in the effects of diet on longevity and/or healthspan. In yeast (*Saccharomyces cerevisiae*), *C. elegans* and *Drosophila*, mutants that over-express Sir2 live longer, and mutants with reduced or no *Sir2* gene function are not sensitive to the effects of diet on lifespan (Kaeberlein et al. 1999; Tissenbaum and Guarente 2001; Rogina and Helfand 2004). However, the view that Sir2 affects lifespan is controversial and has been the subject of recent vigorous challenge based on data indicating that extended lifespan in strains of *C. elegans* tracked not with *Sir2* transgenes but with other loci and that other effects of genetic manipulation used to create long-lived *Sir2* transgenic *Drosophila*, rather than the *Sir2* transgene per se, were responsible for this phenotype (Burnett et al. 2011). However, when Sir2 expression was manipulated in *Drosophila* using an inducible system that eliminated genetic background as a confounding factor, higher Sir2 expression led to longer lifespan (Banerjee et al. 2012).

Lifespan in honeybees is strongly influenced by diet and depends upon whether bees are workers or reproductive queens; workers live an average of less than 1 month in summer conditions when they are foraging, whereas queens live for 2–3 years (Winston 1991). Survival of adult workers is reduced when they are fed diets high in protein (Pirk et al. 2010) but also depends on the dietary source of protein (Altaye et al. 2010). Whether or not Sir2 expression also influences lifespan, and if this influence depends on diet, has not yet been shown in the honeybee. However, an influence of Sir2 on lifespan in the honeybee was indicated indirectly in a recent study of the effect of the plant polyphenol resveratrol, a possible activator of Sir2, on the survival of worker honeybees (Rascon et al. 2012).

In the present study, we used small interfering RNA (siRNA) as a tool to manipulate Sir2 in the honeybee. Gene knock-down using approaches based on RNA interference (RNAi), including use of siRNAs, is now used routinely and is proving to be a useful tool to reduce gene expression in vivo in insects (e.g. Belles 2010; Terenius et al. 2011; Scott et al. 2013). Injection of siRNA into honeybee larvae (Kucharski et al. 2008) or adults (Nelson et al. 2007; Ament et al. 2012) has been effective in reducing expression of specific genes, but less invasive approaches including delivery in nebulised aerosol of siRNA coupled with perfluorocarbon nanoparticles (PFC-NPs) (Li-Byarlay et al. 2013) and in the diet (Jarosch et al. 2011), our chosen route for administration in the current study, have been effective.

Here, we show that that worker honeybees fed diets low in essential amino acids relative to carbohydrate live longer. We also found that a specific (low) ratio of dietary amino acids to carbohydrate (1:500), but not complete absence, elevated the expression of Sir2 after 14 days. We show unequivocally that Sir2 in honeybees is one of the determinants of lifespan when the diet provides amino acids at this ratio by demonstrating a reduction in lifespan under conditions of Sir2 knock-down by siRNA.

## Experimental procedures

### Dietary manipulation in honeybees and measurement of survival

To explore the effect of diet on honeybee lifespan, cohorts of 20 newly emerged *Apis mellifera* adult workers were confined to feed on solutions with defined ratios of essential amino acids and carbohydrates (sucrose) (EAA:C). The bees were removed from two outdoor colonies at Newcastle University originally obtained from stock kept at the National Bee Unit (FERA, Sandhutton, UK). Brood frames were placed in a brood box in a controlled temperature room at 34 °C with 60 % relative humidity and allowed to eclose. Newly emerged workers were brushed off the frame, collected in a large plastic box, and placed in cohorts of 20 in smaller Perspex boxes (11 × 6 × 20 cm). Boxes were held at a constant temperature of 34 °C with 60 % relative humidity in a temperature-controlled room. Feed solutions with defined ratios of essential amino acids and carbohydrates (100 % sucrose and 1:5, 1:10, 1:100, 1:250 and 1:500 EAA:C) were prepared by mixing in the relevant proportions 1 M sucrose and a solution containing equimolar quantities of each of the ten essential amino acids (Met, Try, Arg, Lys, His, Phe, I-Leu, Thr, Leu, Val). Each solution was provided to five boxes of 20 bees. Feed solution was delivered through two modified microcentrifuge tubes with four holes in a line down one side. Each tube was weighed immediately after filling and on each of the following days, before and after replenishing the solution. An adjustment for evaporation was made based on the weight loss measured in feeding tubes in empty boxes. Mortality was recorded each day, and food consumption per bee for all boxes in the experiment was calculated by dividing the adjusted loss in food weight by the number of bees still alive at the point of sampling. We conducted a pilot experiment over 14 days using diets with a range of EAA:C ratios and then conducted an experiment over the full life course using the two diets we found to have the most diverse effects on mortality (1:5 versus 1:500).

### Measurement of Sir2 RNA by reverse transcriptase quantitative PCR

To investigate if any differences in survival between bees on the different diets were accompanied by changes in the expression of Sir2, we measured Sir2 messenger RNA (mRNA) by reverse transcriptase quantitative PCR (RT-qPCR). For the pilot experiment, using all six test diets, measurements were made on three bees taken at day 14 from three separate boxes of bees on each diet. For the experiment conducted over the full life course, using the 1:5 and 1:500 EAA:C diets, nine bees were sampled at days 7 and 14 from one box of 20 bees fed on each diet, separated to the five boxes analysed for survival. Bees were snap-frozen and stored at −80 °C until processing. Whole bees were ground in liquid nitrogen, and RNA was prepared using TRIzol reagent (Invitrogen), following the manufacturer’s instructions. First-strand complementary DNA (cDNA) synthesis was carried out on RNA using Moloney Murine Leukaemia Virus Reverse Transcriptase (Promega), following the manufacturer’s instructions. Quantitative real-time PCR was performed in a Roche LightCycler 480 with 20 μl reactions set up in 96-well format containing LightCycler SYBR Green I Master (Roche), 0.5 μM of each primer (Supplementary Table S2), and 1 μl of cDNA (diluted to 1:4). The reference genes was *A. mellifera* ribosomal protein S8 (Rps8; as in Robertson and Wanner 2006). After denaturing for 5 min at 95 °C, 50 cycles were carried out using the following parameters: 95 °C, 10 s; 55 °C, 10 s; and 72 °C, 15 s. Levels of specific RNAs relative to control, corrected according to levels of reference gene RNAs, were calculated using the ΔΔC_t_ method. PCR products were sequenced (Genevision, UK) to confirm identity to the expected products.

### Effect of Sir2 siRNA on survival

To test whether diet-mediated elevation in Sir2 expression was one of the mechanisms underlying the effect of diet on lifespan, we used siRNAs to knock-down Sir2 in bees fed the 1:500 EAA:C diet. We first tested the efficacy of two siRNA sequences provided at three different concentrations (0.1, 0.05 and 0.025 μg/ml) in the 1:500 diet given to newly emerged workers by measuring Sir2 mRNA levels by RT-qPCR at days 7 and 14. For this experiment, five boxes of 20 bees were maintained under each condition; four boxes were used to monitor survival, and one box was used to sample bees from the preparation of mRNA at days 7 and 14. The diet solution containing siRNA targeted to Sir2 (Sigma; Supplementary Table S1) or control siRNA (Stealth RNAi™ siRNA negative Control LO GC (Invitrogen)) was stored at −20 °C and was replenished in full each day. The maximum knock-down was achieved using siRNA2 at 0.05 μg/ml, which reduced Sir2 mRNA levels by 67 % at day 7 and by 48 % at day 14 compared with bees fed a control siRNA (Supplementary Fig. S2A, B). This same concentration was also optimal for siRNA1, but this siRNA knock-down—of 31 %—reached a statistical significance only at day 14. Bees fed siRNA2 at 0.05 μg/ml had higher mortality over the 14-day experiment than bees fed the control siRNA or siRNA1 (Supplementary Fig. S2C), indicating an effect of the parallel reduction in Sirt2 expression on lifespan that we then tested over the full lifespan. We thus proceeded to use both siRNAs at 0.05 μg/ml in the 1:500 EAA:C diet to achieve knock-down of Sir2 in bees fed the siRNA from the point of eclosure until death. Six boxes of 20 bees were maintained under each condition (control siRNA, siRNA1, siRNA2). Survival and food consumption was measured in five boxes, and the sixth box was used to sample bees at days 7 and 14 for the preparation of RNA to confirm efficacy of Sir2 knock-down.

### Statistical analysis of data

Survival data were analysed using a Cox regression (Coxreg) analysis with diet and cohort as a covariates in SPSS (IBM SPSS Statistics 19). Time of a death event over the period of study was entered as the dependent variable in the model where ‘time of death’ was defined as the death of a single bee in each cohort (replicate). Comparisons between groups were evaluated using the ‘indicator’ contrasts in SPSS. Data on expression of mRNA, measured by RT-qPCR, were analysed by Student’s unpaired *t* test or by one-way ANOVA followed by Dunnett’s pairwise post hoc tests. Food consumption data (energy intake) were analysed using SPSS (IBM SPSS Statistics 19) with diet as a main effect and cohort as a random effect in a two-way ANOVA or with diet, age and cohort in a three-way ANOVA; differences in food consumption were measured by pairwise, least squares difference (lsd) post hoc tests.

## Results

### Dietary amino acid-to-carbohydrate concentration affects honeybee survival

The pilot experiment to explore the effect of a range of different dietary EAA:C ratios on survival revealed that bees fed a 1:5 diet had higher mortality than bees on all other diets we tested. Bees fed the 1:500 diet lived longer than bees fed pure sucrose or the 1:10 diet over a 14-day period (Supplementary Fig. S1A). Dietary EAA:C ratio did not influence total energy intake (two-way ANOVA, diet main effect, *F*_5,20_ = 0.778, *P* = 0.577, Supplementary Table S3).

**Fig. 1:**
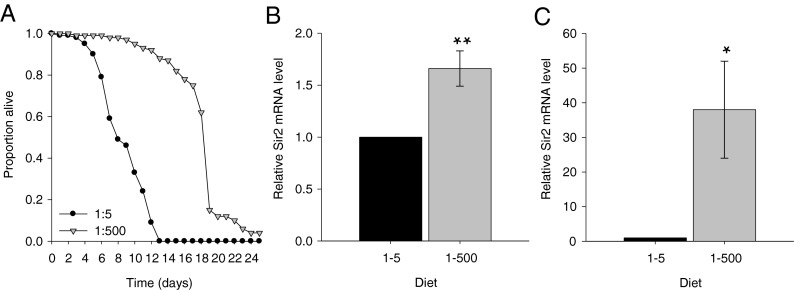
Essential amino acid-to-carbohydrate ratio affects survival and Sir2 expression. **a** Bees fed diets high in the concentration of EAAs (1:5 EAA to sucrose) had reduced survival. Data are expressed as the proportion of bees alive on each day for *n* = 100 (five cohorts of 20 bees) for each condition. **b**, **c** Relative levels of expression of Sir2 mRNA measured by RT-qPCR in bees given the different diets sampled at day 7 (**b**) and day 14 (**c**). Data are mean ± SEM for *n* = 9, normalised to Rps8. *P < 0.05; **P < 0.01, compared with sucrose by Student’s unpaired *t* test

In the follow-up independent experiment conducted over the full lifespan to compare the effects of the 1:500 and 1:5 diets, we found that honeybees fed on the high amino acid diet (1:5) were 30 times more likely to die prematurely than those fed the 1:500 diet [Fig. 1a; Coxreg, 1:5 × 1:500, *χ*_1_^2^ = 84.8, hazard ratio (HR) = 30 (95 % confidence interval (CI) 14–63), *P* < 0.001]. Cohort did not influence the risk of mortality [Coxreg, *χ*_1_^2^ = 2.38, HR = 1.09 (95 % CI 0.97–1.12), *P* = 0.112].

### Dietary essential amino acid-to-carbohydrate balance affects expression of Sir2

In the pilot experiment where all diets were compared, only the 1:500 diet elevated Sir2 mRNA at day 14 (one-way ANOVA, *F*_4,5_ = 18.3, *P* = 0.013; Supplementary Fig. S1B). This same observation was replicated in the independent experiment we carried out over the full life course, where Sir2 mRNA was higher at both days 7 and 14 in bees fed the 1:500 diet compared with the 1:5 diet (*P* < 0.01 and *P* < 0.05, respectively, by Student’s unpaired *t* test; Fig. 1b). Thus, bees fed the 1:500 EAA:C diet not only survived longer than bees fed pure sucrose or diets with a higher amino acid content, but they also exhibited elevated transcript levels of Sir2.

### Knock-down of Sir2 expression shortens lifespan

**Fig. 2:**
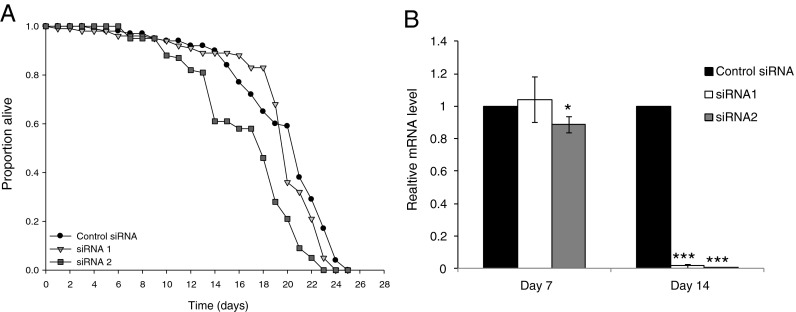
Knock-down of Sir2 expression reduces lifespan. **a** Bees fed the 1:500 diet died at a faster rate when Sir2 was knocked down using siRNA2. Use of siRNA1 did not reduce survival compared to the control siRNA. (*N* = 5 cohorts of 20 bees/cohort) for each condition (Supplementary Table S5). **b** Relative levels of expression of Sir2 mRNA measured by RT-qPCR to confirm efficacy of siRNA-mediated knock-down in bees sampled at days 7 and 14. Data are mean ± SEM for *n* = 9, normalised to Rps8 for day 7 and based only on Sir2 amplification from equal quantities of cDNA for day 14, since both Rps8 became unstable and varied substantially between samples. *P < 0.05; ***P < 0.001, compared with control by one-way ANOVA then a Dunnett’s post hoc test

Knock-down of Sir2 using siRNA in bees fed the 1:500 EAA:C diet significantly reduced lifespan (Coxreg, *χ*_2_^2^ = 39.1, *P* < 0.001). Cohort did not influence the risk of mortality [Coxreg, replicate, *χ*_1_^2^ = 0.80, HR = 1.04 (95 % CI 0.95–1.15), *P* = 0.380]. Pairwise contrasts of the control siRNA against the two siRNAs targeted to Sir2 revealed that siRNA2 significantly increased the instantaneous risk of mortality by a factor of 2.2 [Fig. 2a and Supplementary Table S4; Coxreg, siRNA2 × control siRNA, *χ*_1_^2^ = 33.3, HR = 2.5 (95 % CI 1.8–3.4), *P* < 0.001]. Bees fed siRNA1 had a 1.2 greater risk of mortality, but this risk was not significantly different from the control group [Fig. 2a and Supplementary Table S5; Coxreg, siRNA1 × control siRNA, *χ*_1_^2^ = 1.98, HR = 1.2 (95 % CI 0.91–1.7), *P* = 0.159]. siRNA2 was also more effective than siRNA1 at knock-down of Sir2; siRNA2 reduced Sir2 mRNA levels by 11 % at day 7, at which point there was no effect of siRNA1 (Fig. 2b). Both siRNAs reduced Sir2 mRNA dramatically (by 84 % for siRNA1 and by 96 % for siRNA2) at day 14 (Fig. 2b). This difference in efficacy may be the reason that siRNA1 did not appreciably affect survival. Bees fed the diet containing the control siRNA ate slightly less than those fed either of the siRNAs that targeted Sir2 (Supplementary Fig. S3).

## Discussion

Our data establish two important novel findings: first, we show that the dietary balance of EAAs and carbohydrates can affect Sir2 expression. Sir2 was especially elevated on a diet with low but not absent EAAs. Second, we demonstrate that siRNA knock-down of Sir2 reduces lifespan. We additionally show that diets high in EAAs shorten lifespan, albeit through mechanisms independent of Sir2.

The interactions of amino acids with other dietary components to influence lifespan and reproduction are complex and likely to depend on the relative ratios of specific macronutrients, including EAAs and carbohydrates (reviewed in Piper et al. 2011; Simpson and Raubenheimer 2012). Previous experiments on *Drosophila* using diets high in amino acid had a ratio, which we calculated from the information provided, of 1:1–1:2 (Grandison et al. 2009) or 1:9 (Sun et al. 2012). In our experiments, diets high in EAAs (e.g. the 1:5 diet) had a small effect on survival during the first 7 days but had much greater effect during the last 7 days of the first experiment. This rapid change in the ability to tolerate dietary amino acids may reflect a physiological change in the ability to use dietary EAAs when worker bees undergo transition from the caste of nurse bee to that of forager. Nurses undergo a suite of physiological and behavioural changes that prepare them for foraging, including a reduction in the fat body (Seehuus et al. 2007; Chan et al. 2011) and loss of the ability to digest protein (Crailsheim 1986; Moriz and Crailsheim 1987; Szolderits and Crailsheim 1993). These changes are orchestrated by feedback in the levels of juvenile hormone and vitellogenin (Robinson 1987; Mutti et al. 2011; Antonio et al. 2008), which in turn are affected by exposure to the queen bee’s mandibular pheromone (Corona et al. 2007; Dietz et al. 1979).

In addition to showing that Sir2 was elevated in bees fed the 1:500 diet and that knock-down of Sir2 in bees fed this diet reduced lifespan, we show that worker honeybees have a much increased risk of mortality from consuming diets high in EAAs. This was also corroborated in another, separate study of the influence of EAAs in diet on nutrient balancing in worker honeybees (Paoli et al. 2014). These data could suggest that excess EAAs become toxic, possible because bees cannot make use of excess amino acids as fuel, excrete excess amino acids or process excess nitrogenous waste after catabolism. Forager bees require large amounts of haemolymph hexoses to fuel flight (Suarez et al. 1996, 2005) and have optimized their metabolism to produce sufficient ATP (Kunieda et al. 2006). They can use proline (Suarez et al. 2005) but, unlike other animals, must be less able to use other EAAs for fuel. The inability to use amino acids to make ATP could be the result of an evolutionary trade-off forced by the optimization of the use of hexoses as fuel (Suarez et al. 1996, 2005). It may also be driven by selection towards resource partitioning in colonies such that protein resources are reserved for the larvae and the queen and are not used to support sterile, foraging workers (Amdam and Omholt 2002).

We can attribute the effect of the different diets on honeybee lifespan to the balance of EAAs to carbohydrate and exclude an influence of caloric intake on the basis that energy intake did not differ between the 1:500 diet and any of the other diets.

Previous studies in *Drosophila* have shown that, in contrast to diets high in amino acids, diets diluted in amino acids (yeast) extend lifespan (Bass et al. 2007; Broughton et al. 2010; Banerjee et al. 2012) through mechanisms likely to be pleiotropic, including *Drosophila* insulin-like peptide (DILP) neurons in the brain (Broughton et al. 2010) and target of rapamycin (TOR) signalling (Geminard et al. 2009). Like the bees in our study fed the 1:500 diet, flies fed on yeast-diluted diets also expressed more Sir2 and had longer lifespans (Banerjee et al. 2012).

Our observations show that Sir2 contributes to, yet is not the only determinant of, differences in lifespan on the diets we tested. The reduction in lifespan we observed in bees fed the 1:500 EAA:C diet when Sir2 expression was reduced by siRNA demonstrates that Sir2 contributes to longevity under these conditions. However, the 1:500 EAA:C diet was unique among those tested in increasing Sir2 expression yet mortality was lower on other diets lower in protein than the 1:5 EAA:C revealing that other mechanisms are important in determining lifespan under other dietary regimes. The contribution of other mechanisms is also clear from the fact that Sir2 knock-down on the 1:500 EAA:C diet did not curtail lifespan extent observed with the 1:5 EAA:C diet.

One of two siRNAs targeted to Sir2 that we used (siRNA1) had only a marginal effect on lifespan yet, by day 14, had dramatically reduced Sir2 expression. In all experiments, this siRNA was the less effective of the two and, in contrast with the other siRNA (siRNA2), did not reduce Sir2 mRNA levels significantly at day 7. It is thus possible that siRNA1 became effective only when very close to the end of the lifespan, and its impact on lifespan was, therefore, difficult to measure. The concept that early life nutrition in particular can affect long-term health and survival is well established in mammals (e.g. reviewed in Aiken and Ozanne 2013), and evidence is emerging to support the view that the same phenomenon applies to insects (Buescher et al. 2013). Sir2 knock-down resulted in a slight elevation of food consumption. In contrast, other studies reported that effects of Sirt1/Sir2 or downstream signalling events in the brain on food intake reduce food intake in mammals and *Drosophila* (Dietrich et al. 2010; Hong et al. 2012). The apparent discord could be a species-specific effect or related to the fact that the background diet we used was the one that supported maximum lifespan, but this is an observation that is currently difficult to reconcile with these other findings without further investigation.

**Fig. 3:**
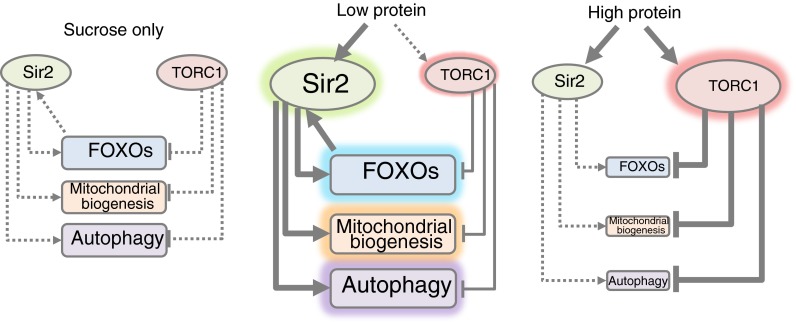
A schematic representation of proposed effects of different levels of dietary protein on signalling through Sir2 and TORC1 with effects on mediators of lifespan. Activation of FOXO transcription factors, mitochondrial biogenesis and autophagy are known to promote longevity. *Size of symbols* indicates a relative level of activation, and activation is also indicated by *shaded highlighting round* the relevant symbol. *Arrow heads* indicate activating effects; inhibitory effects are indicated by *lines ending in vertical bars*. Protein (amino acids) activates both Sir2 (a new proposal) and TORC1 (established). With low protein, where activation of TORC1 is to only a low level, increased Sir2 expression leads to FOXO activation, and without substantial TORC1-mediated inhibition of FOXOs, the autoregulatory positive feedback loop between Sir2 and FOXO1 is active, thus further activating Sir2 transcription. At higher protein levels, TORC1 is highly activated, leading to repression of mitochondrial biogenesis and of autophagy. Moreover, inhibition of FOXO interrupts the positive Sir2 feedback loop; thus, Sir2 levels fall

We propose a scheme (Fig. 3) in which the response to dietary EAAs depends on their concentration. Low concentrations of EAAs support a longer lifespan in honeybees through Sir2 signalling, whereas high concentrations of EAAs curtail lifespan through TOR signalling and other mechanisms. TOR is a nutrient-sensing protein kinase that regulates growth and protein synthesis, and TOR activation is associated with curtailed lifespan (Bjedov et al. 2010; Kapahi et al. 2010; Zid et al. 2009). TOR signalling—specifically the functional TOR complex TORC1—is activated by amino acids (Zoncu et al. 2011). We propose that only high concentrations of EAAs can activate TOR to a level that affects lifespan. Our data clearly show that low levels of EAAs increase Sir2 expression. The pleiotropic effects of Sir2 activation to extend lifespan include deacetylation and, thus, activation of the transcription factor FOXO (Brunet et al. 2004). We propose that a positive autoregulatory feedback loop that exists between FOXO and (mammalian) Sirt1 (Xiong et al. 2011) is also active in the honeybee. Sir2 gene transcription is activated by FOXO; thus, activation of FOXO by Sir2-mediated deacetylation further increases Sir2 gene expression accounting for the very high levels of Sir2 mRNA we observed in bees fed the 1:500 EAA:C diet. FOXO is repressed by TOR signalling, as revealed by recent work in *C. elegans* (Robida-Stubbs et al. 2012). We do not propose that this scheme is exhaustive with respect to the likely pleiotropic effects of Sir2 on lifespan.

Our observations further clarify that sirtuins can play a direct role in modifying lifespan and add support to the view that sirtuins are potential targets for nutritional or pharmacological interventions to increase human lifespan or healthspan.

## Electronic supplementary material

Below is the link to the electronic supplementary material.

Supplementary Fig. S1 Essential amino acid to carbohydrate ratio affects survival and Sir2 expression. (A) Newly-emerged adult worker honeybees were fed with 6 different diets varying in the EAA:C ratio for 14 days. The EAA:C ratio affected bee lifespan. Bees fed diets high in EAAs had a greater risk of dying than those fed diets low in EAAs or sucrose alone (Coxreg, *χ*_5_^2^ = 449, *P* < 0.001). Bees fed the 1:500 diet had 3.4 times lower instantaneous risk of dying than bees fed sucrose alone (Coxreg, sucrose vs 1:500 diet, *χ*_1_^2^ = 4.69, HR = 0.29 [95 % CI 0.09–0.88], *P* = 0.030). Bees fed the 1:10 diet had a 3.3 times greater risk of dying than those fed sucrose (Coxreg, *χ*_1_^2^ = 13.8, HR = 3.3 [95 % CI 1.7–6.3], *P* < 0.001) whereas those fed the 1:5 diet had a 15 times greater risk of dying over the 14 day period than the sucrose control (Coxreg, *χ*_1_^2^ = 81.1, HR = 15 [95 % CI 8.3–27], *P* < 0.001). All other treatments did not significantly change the risk of dying compared with sucrose alone (Coxreg, all *P* > 0.05). (B) Diet had a significant effect on the expression of Sir2 at day 14 (1-way ANOVA, *F*_4,10_ = 18.3, *P* < 0.001). Bees fed the 1:500 diet had over 10 times the transcript levels of Sir2 of bees fed diets of sucrose, 1:5, 1:100 and 1:250 EAA:C (lsd, *P*_suc_ < 0.001, *P*_1:5_ < 0.001, *P*_1:100_ < 0.001, *P*_1:250_ < 0.001). Sir2 levels in bees fed the diets with 1:5, 1:100 and 1:250 EAA:C were not significantly different to levels in bees fed sucrose only (lsd, *P*_1:5_ = 0.870, *P*_1:100_ = 0.861, *P*_1:250_ = 0.926). (PPT 176 kb)

Supplementary Fig. S2 Knockdown of Sir2 in honeybees by siRNA. Relative levels of expression of Sir2 mRNA measured by RT-qPCR to establish efficacy of siRNA-mediated knock-down of one of two different siRNAs or a control siRNA administered in the 1:500 diet at the concentrations shown in bees sampled at (A) day 7 and (B) day 14. Data are the mean ± SEM for *n* = 3, normalised to Rps8. *P < 0.05, **P < 0.01 compared with control by 1-way ANOVA then Dunnett’s post hoc test. (C) Survival over 14 days of bees fed the three different siRNAs in the 1:500 diet at the concentrations shown. Data are for *n* = 80 (4 boxes of 20 bees) for each condition. (PPT 153 kb)

Supplementary Fig. S3 Total food consumption under conditions of Sir2 knockdown by siRNA. siRNA treatment and age (days 0–7 or 7–14 days) affected the amount of solution consumed by bees (3-way ANOVA, age*diet, *F*_4,16_ = 7.61, *P* = 0.001). Cohort did not have a significant effect on consumption (3-way ANOVA, cohort, *F*_4,16_ = 1.46, *P* = 0.350). Bees fed siRNA 1 and 2 consumed similar amounts of diet (lsd, *P* = 0. 8269), but both groups of bees receiving siRNA 1 or siRNA2 consumed more than the control siRNA group (lsd, *P*_siRNA1_ = 0.007, *P*_siRNA2_ = 0.004). Data are for *n* = 100 (5 cohorts of 20 bees) for each condition, based on measurements made each day over the full experiment. Food consumption was not measured beyond 14 days. (PPT 104 kb)

ESM 4 (DOC 128 kb)
