# Supplementary figures and images for: The dietary proportion of essential amino acids and Sir2 influence lifespan in the honeybee
Source: Age (Dordr). 2014 Apr 10;36(3):9649. doi: 10.1007/s11357-014-9649-9 (PMC4082578; doi:10.1007/s11357-014-9649-9)

## Slide 1
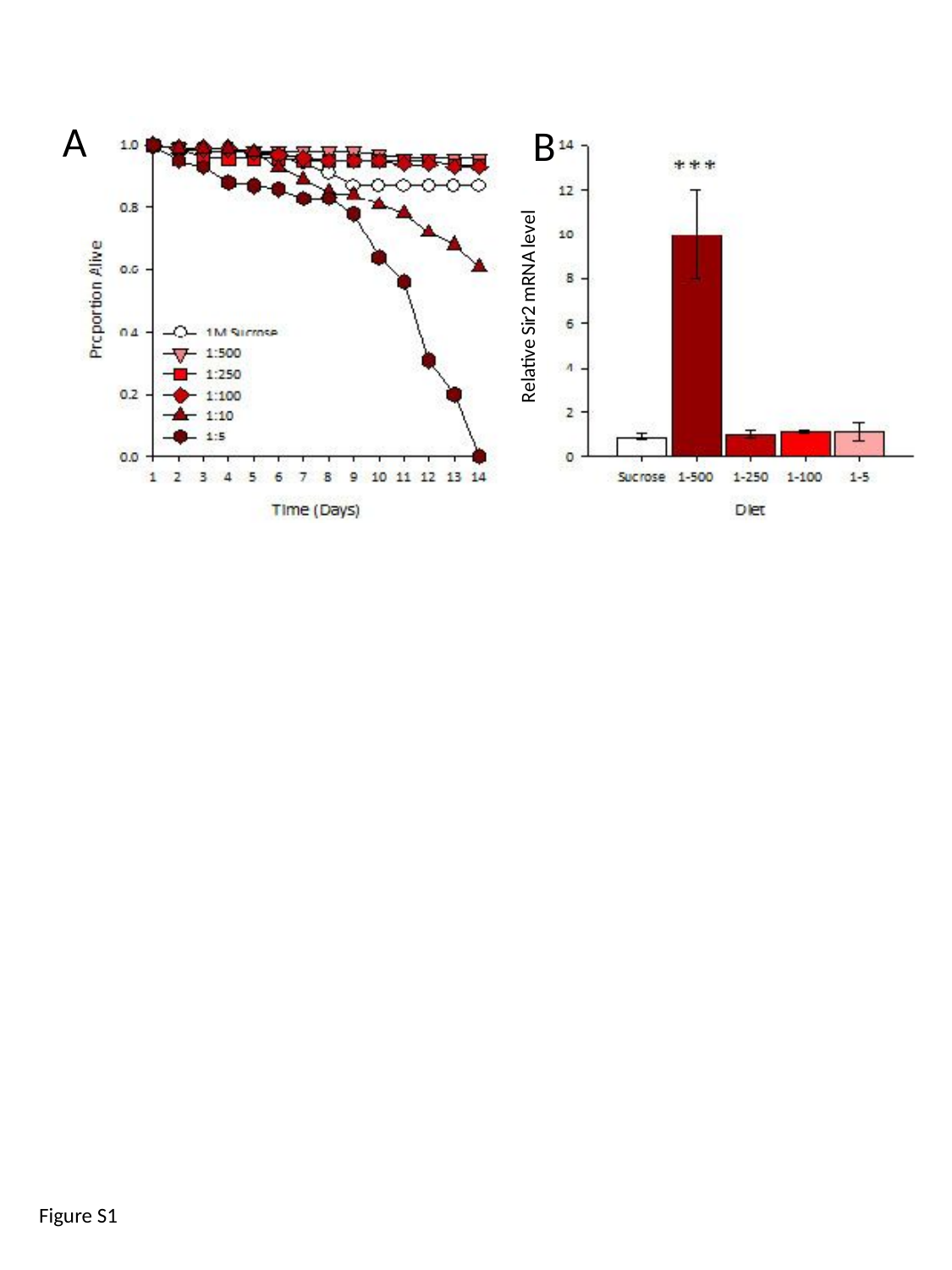

Figure S1

Supplement: Supplementary file 1 — Essential amino acid to carbohydrate ratio affects survival and Sir2 expression. (A) Newly-emerged adult worker honeybees were fed with 6 different diets varying in the EAA:C ratio for 14 days. The EAA:C ratio affected bee lifespan. Bees fed diets high in EAAs had a greater risk of dying than those fed diets low in EAAs or sucrose alone (Coxreg, χ 5 2 = 449, P < 0.001). Bees fed the 1:500 diet had 3.4 times lower instantaneous risk of dying than bees fed sucrose alone (Coxreg, sucrose vs 1:500 diet, χ 1 2 = 4.69, HR = 0.29 [95 % CI 0.09–0.88], P = 0.030). Bees fed the 1:10 diet had a 3.3 times greater risk of dying than those fed sucrose (Coxreg, χ 1 2 = 13.8, HR = 3.3 [95 % CI 1.7–6.3], P < 0.001) whereas those fed the 1:5 diet had a 15 times greater risk of dying over the 14 day period than the sucrose control (Coxreg, χ 1 2 = 81.1, HR = 15 [95 % CI 8.3–27], P < 0.001). All other treatments did not significantly change the risk of dying compared with sucrose alone (Coxreg, all P > 0.05). (B) Diet had a significant effect on the expression of Sir2 at day 14 (1-way ANOVA, F 4,10 = 18.3, P < 0.001). Bees fed the 1:500 diet had over 10 times the transcript levels of Sir2 of bees fed diets of sucrose, 1:5, 1:100 and 1:250 EAA:C (lsd, P suc < 0.001, P 1:5 < 0.001, P 1:100 < 0.001, P 1:250 < 0.001). Sir2 levels in bees fed the diets with 1:5, 1:100 and 1:250 EAA:C were not significantly different to levels in bees fed sucrose only (lsd, P 1:5 = 0.870, P 1:100 = 0.861, P 1:250 = 0.926). (PPT 176 kb) [file 11357_2014_9649_MOESM1_ESM.ppt]

## Slide 1
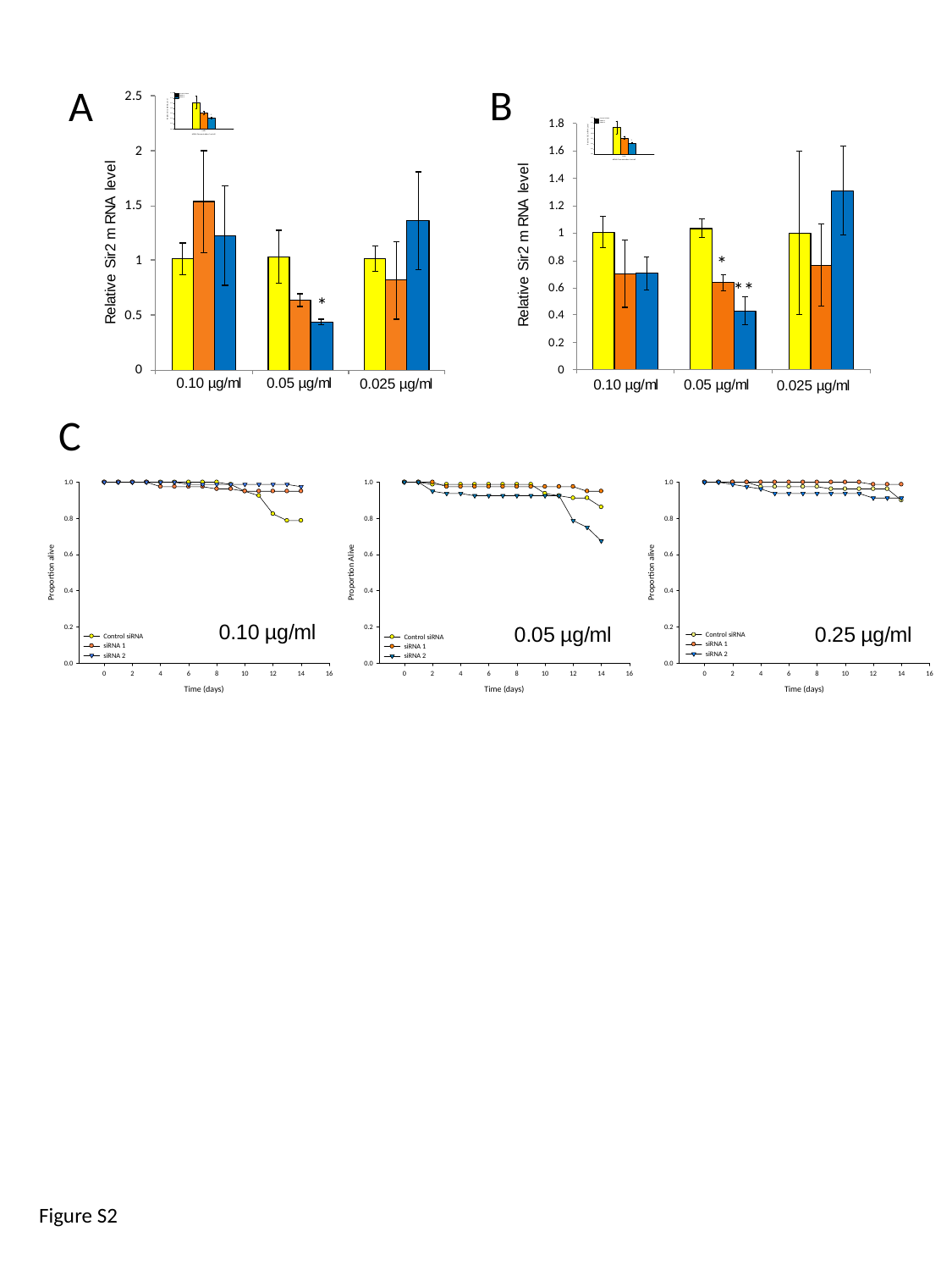

Figure S2

Supplement: Supplementary file 2 — Knockdown of Sir2 in honeybees by siRNA. Relative levels of expression of Sir2 mRNA measured by RT-qPCR to establish efficacy of siRNA-mediated knock-down of one of two different siRNAs or a control siRNA administered in the 1:500 diet at the concentrations shown in bees sampled at (A) day 7 and (B) day 14. Data are the mean ± SEM for n = 3, normalised to Rps8. *P < 0.05, **P < 0.01 compared with control by 1-way ANOVA then Dunnett’s post hoc test. (C) Survival over 14 days of bees fed the three different siRNAs in the 1:500 diet at the concentrations shown. Data are for n = 80 (4 boxes of 20 bees) for each condition. (PPT 153 kb) [file 11357_2014_9649_MOESM2_ESM.ppt]

## Slide 1
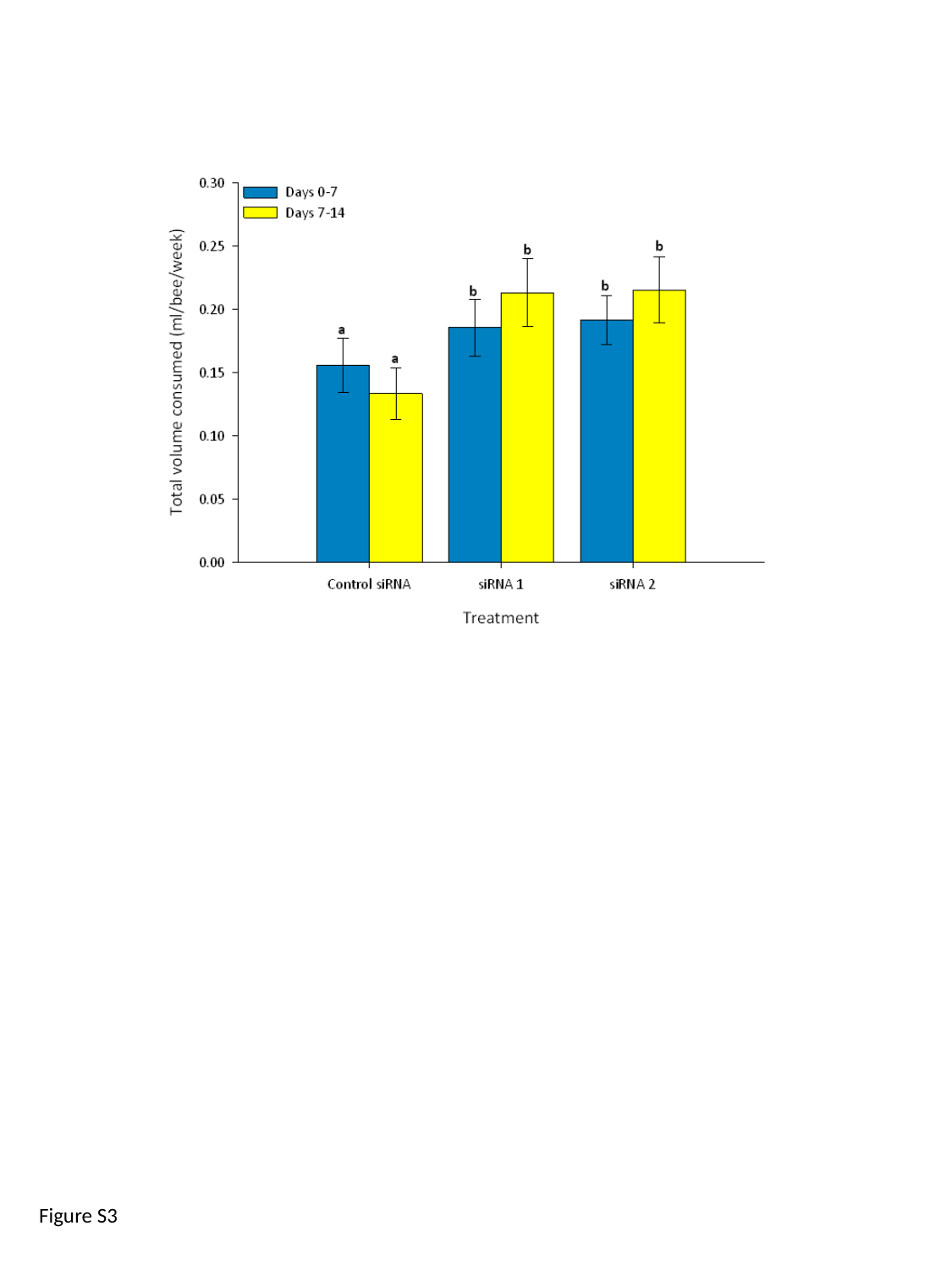

Figure S3

Supplement: Supplementary file 3 — Total food consumption under conditions of Sir2 knockdown by siRNA. siRNA treatment and age (days 0–7 or 7–14 days) affected the amount of solution consumed by bees (3-way ANOVA, age*diet, F 4,16 = 7.61, P = 0.001). Cohort did not have a significant effect on consumption (3-way ANOVA, cohort, F 4,16 = 1.46, P = 0.350). Bees fed siRNA 1 and 2 consumed similar amounts of diet (lsd, P = 0. 8269), but both groups of bees receiving siRNA 1 or siRNA2 consumed more than the control siRNA group (lsd, P siRNA1 = 0.007, P siRNA2 = 0.004). Data are for n = 100 (5 cohorts of 20 bees) for each condition, based on measurements made each day over the full experiment. Food consumption was not measured beyond 14 days. (PPT 104 kb) [file 11357_2014_9649_MOESM3_ESM.ppt]
