# Supplementary material for: The dietary proportion of essential amino acids and Sir2 influence lifespan in the honeybee
Source: Age (Dordr). 2014 Apr 10;36(3):9649. doi: 10.1007/s11357-014-9649-9 (PMC4082578; doi:10.1007/s11357-014-9649-9)
Supplement: Supplementary file 4 — (DOC 128 kb) [file 11357_2014_9649_MOESM4_ESM.doc]

**Table S1.** siRNA sequences. Sequences are in the 5’-3’ direction and subscript numerals refer to positions within the sequences deposited under the stated Genbank accession number.

| **Gene** | **Gene ID** | **siRNA** | **Sequence _(position in sequence)_ 5’-3’** |
| --- | --- | --- | --- |
| Sir2 histone deacetylase, transcript variant 1 (*Sir2*) | XM_003251332.1 | siRNA1 | _1907_gaaaucucuucggaaagugcauua-uu_1932_ |
|  |  | siRNA2 | _2268_auucagAuccugaagaauacgauca-uu_2294_ |

**Table S2.** PCR primers. Sequences are in the 5’-3’ direction and subscript numerals refer to positions within the sequences deposited under the stated Genbank accession numbers. Forward (5’) then reverse (3’) primer sequences are stated in each case.

| **Gene** | **Gene ID** | **Sequence _(position in sequence)_ 5’-3’** | **Product length** |
| --- | --- | --- | --- |
| Sir2 histone deacetylase, transcript variant 1 (*Sir2*) | XM_003251332.1 | _2100_gttgtaatagtgttgacagtcacgaagac_2119_ | 184 |
|  |  | _2274_tctgaatatacttctgctccagg_2252_ |  |
| Ribosomal protein S8 (*Rps8*) | NM_001011604.3 | _376_acgaggtgcgaaactgactgaagc_399_ | 182 |
|  |  | _557_cacgaccgcactgtccaggt_538_ |  |

**Table S3**. Diet composition, energy content and intake by bees of diets with different ratios of essential amino acids to carbohydrate. Data were analysed using 2--way ANOVA with diet as a main effect and cohort as a random effect. Differences in energy intake compared with the 1:500 diet were measured by pairwise, least squares difference *post hoc* tests.

| **Diet** | Sucrose | 1:500 | 1:250 | 1:100 | 1:10 | 1:5 |
| --- | --- | --- | --- | --- | --- | --- |
| **[Sucrose] (mg/ml)** | 342.30 | 342.30 | 342.30 | 342.30 | 342.30 | 342.30 |
| **kcal/ml from sucrose** | 1.369 | 1.369 | 1.369 | 1.369 | 1.369 | 1.369 |
| **[Glycine] (mg/ml)** | 0 | 0.0298 | 0.0596 | 0.149 | 1.49 | 2.98 |
| **[Tryptophan] (mg/ml)** | 0 | 0.0408 | 0.0816 | 0.204 | 2.04 | 4.08 |
| **[Arginine] (mg/ml)** | 0 | 0.0348 | 0.0696 | 0.174 | 1.74 | 3.48 |
| **[Lysine] (mg/ml)** | 0 | 0.0292 | 0.0584 | 0.146 | 1.46 | 2.92 |
| **[Histidine] (mg/ml)** | 0 | 0.031 | 0.062 | 0.155 | 1.55 | 3.10 |
| **[Phenylalanine] (mg/ml)** | 0 | 0.033 | 0.066 | 0.165 | 1.65 | 3.30 |
| **[Isoleucine] (mg/ml)** | 0 | 0.0262 | 0.0524 | 0.131 | 1.31 | 2.62 |
| **[Threonine] (mg/ml)** | 0 | 0.0238 | 0.0476 | 0.119 | 1.19 | 2.38 |
| **[Leucine] (mg/ml)** | 0 | 0.0262 | 0.0524 | 0.131 | 1.31 | 2.62 |
| **[Valine] (mg/ml)** | 0 | 0.0234 | 0.0468 | 0.117 | 1.17 | 2.34 |
| **Total [EAA] (mg/ml)** | 0 | 0.2982 | 0.596 | 1.491 | 14.91 | 29.82 |
| **Kcal/ml from EAA** | 0 | 0.00119 | 0.00238 | 0.00596 | 0.0596 | 0.119 |
| **Total kcal/ml** | 1.369 | 1.370 | 1.371 | 1.375 | 1.429 | 1.488 |
| **Daily consumption per bee (mean ± SEM) (ml)** | 0.014 ± 0.001 | 0.016 ± 0.001 | 0.015 ± 0.003 | 0.014 ± 0.001 | 0.018 ± 0.004 | 0.018 ± 0.001 |
| **Mean energy intake per bee per day (mean ± SEM) (kcal)** | 0.020± 0.001 | 0.022 ± 0.001 | 0.021 ± 0.004 | 0.020 ± 0.002 | 0.026 ± 0.005 | 0.026 ± 0.001 |
| **P versus 1:500 diet** | 0.633 | N/A | 0.814 | 0.626 | 0.392 | 0.120 |

**Table 54**. Survival data for bees fed diets with a ratio of essential amino acids to carbohydrate of 1:5 or 1:500. Each box contained 20 bees at the start of the experiment; numbers show the number of live bees in each box on each day of the experiment. Each of the five experiments for each diet was run independently.

**Table S5**. Survival data for bees fed the 1:500 diet containing different siRNAs. Each box contained 20 bees at the start of the experiment; numbers show the number of live bees in each box on each day of the experiment. Each of the five experiments for each condition was run independently.
